# Berberine suppresses mechanical stretch-induced apoptosis and proliferation in vascular smooth muscle cells via ROS/PDI/Drp1 signaling pathway

**DOI:** 10.3389/fphar.2026.1921727

**Published:** 2026-09-25

**Authors:** Ning Lin, Haiming Niu, Xiaodong Cai, Shuying Liu, Miaolian Chen, Conghui Yu, Yingzhang Cai, Linli Wang

**Affiliations:** 1 Key laboratory of Functional and Clinical Translational Medicine, Fujian province, Xiamen Medical College, Xiamen, China; 2 Department of Critical Care Medicine, Zhongshan People’s Hospital, Zhongshan, China; 3 Zhongshan School of Medicine, Sun Yat-sen University, Guangzhou, China; 4 Department of Traditional Chinese Medicine, Zhongshan People’s Hospital, Zhongshan, China; 5 Department of Cardiology, The Third Affiliated Hospital of Sun Yat-sen University, Guangzhou, China

**Keywords:** apoptosis, berberine, heterogeneity, proliferation, stretch stress, vascular smooth muscle cell

## Abstract

**Background:**

Vascular smooth muscle cells (VSMCs) exhibit heterogeneous responses to mechanical stretch stress (SS), with some undergoing apoptosis and others proliferating. The mechanisms underlying this differential fate decision remain unclear.

**Objective:**

This study investigated whether SS-induced heterogeneity in F-actin expression dictates distinct mitochondrial reactive oxygen species (ROS) responses, leading to different VSMC fates, and evaluated the therapeutic potential of the berberine (BBR).

**Methods:**

VSMCs were subjected to SS. F-actin heterogeneity was characterized, and mitochondrial ROS (mtROS), proliferation, and apoptosis were assessed. The involvement of the PKCα/PDI/Drp1 signaling axis was examined using pharmacological inhibitors and BBR.

**Results:**

SS induced heterogeneous mtROS production in VSMCs with varying F-actin levels. This differential ROS signaling concurrently activated the pro-proliferative mTOR/p70 pathway and the pro-apoptotic caspase-9/3 cascade via the PKCα/PDI/Drp1 axis. Importantly, BBR treatment effectively attenuated both SS-induced proliferation and apoptosis by suppressing PKCα activation and subsequent mitochondrial signaling.

**Conclusion:**

Our findings reveal that F-actin heterogeneity determines VSMC fate under SS by driving distinct mitochondrial ROS responses. BBR, by counteracting these ROS-dependent pathways, emerges as a promising therapeutic strategy for managing stretch-related vascular pathologies, such as hypertension-induced atherosclerosis.

## 1 Introduction

Hypertension is a predominant risk factor for the development and progression of atherosclerosis (AS). The pathological mechanical forces associated with hypertension can be categorized into shear stress, which acts on the vascular endothelium, and stretch stress (SS), which acts perpendicularly on the vascular wall. Shear stress primarily modulates endothelial cell function, whereas SS affects multiple vascular layers, including endothelial cells, vascular smooth muscle cells (VSMCs), and adventitial fibroblasts (Li et al., 2026). VSMCs are crucial cells for maintaining vascular elasticity, structural integrity, and intercellular signaling, and their dysfunction—characterized by aberrant proliferation and apoptosis-is a critical event in the initiation and progression of AS (Frismantiene et al., 2018). However, how SS precisely orchestrates these opposing VSMC fates remains unclear.

Many studies have shown that stretch stress is a key factor in vein graft failure, where simultaneous proliferation and apoptosis of VSMCs occur within arteriosclerotic lesions. *In vitro* studies have further revealed heterogeneity among VSMCs under SS stimulation. Our preliminary data indicated that SS induces heterogeneous ROS production in VSMCs, correlating with distinct levels of F-actin expression. These observations indicate heterogeneity among individual VSMCs; however, whether this heterogeneity mediates the simultaneous increase in proliferation and apoptosis, and the mechanisms involved, remain to be elucidated.

Mitochondria, as the primary site of cellular ROS production, are crucial for regulating ROS levels, cell proliferation, and apoptosis (Ping et al., 2017). In endothelial cells and neurons, protein disulfide isomerase (PDI) modulates mitochondrial fission by interacting with mitochondrial dynamin-related protein 1 (Drp1) (Lee and Kim, 2018), influencing mitochondrial ROS production and downstream signaling pathways. PDI functions as a thiol reductase for Drp1, regulating its redox status and mitochondrial fission activity (Kim et al., 2018). Drp1-mediated mitochondrial fission plays a critical role in vascular smooth muscle remodeling and other cardiovascular diseases (Jin et al., 2021). It has been reported that the PDI/MAPK redox system mediates SS-induced simultaneous increases in VSMC proliferation and apoptosis (Wang L. et al., 2020). Moreover, PKC-α regulates endoplasmic reticulum (ER) stress by modulating intracellular Ca^2+^ levels (Wang G. et al., 2020) and contributes to maintaining the proliferative-apoptotic equilibrium (Wang L. et al., 2020). These findings suggest that SS may induce mitochondrial dysfunction via the PKC-α/PDI/Drp1 signaling axis, resulting in heterogeneous ROS production and altered VSMC phenotypic responses.

In this study, live-cell F-actin staining was used to characterize VSMC heterogeneity, and ROS co-staining experiments were performed to investigate the underlying mechanisms. It was hypothesized that SS-induced mitochondrial alterations in VSMCs with heterogeneous F-actin expression lead to differential ROS production via the PKCα/PDI/Drp1 axis, simultaneously promoting proliferation and apoptosis, and that BBR can reduce these effects. These findings provide novel mechanistic insights into hypertension-induced vascular remodeling and identify potential therapeutic targets to prevent and treat hypertension-associated vascular pathology.

## 2 Materials and methods

### 2.1 Materials

Primary antibodies: SM-α-actin (Abcam, ab124964), PDI (Abcam, ab2792), Drp1 (Cell Signaling Technology, 8570S), Ki67 (Novus, NBP2-22112), P-PKCα (Cell Signaling Technology, 9375), PKCα (Cell Signaling Technology, 59,754), P-mTOR (Cell Signaling Technology, 5536S), mTOR (Cell Signaling Technology, 2983S), P-p70 (Cell Signaling Technology, 9204S), p70 (Cell Signaling Technology, 2780S), Caspase-9 (Abcam, ab202068), Cleaved-Caspase-9 (Cell Signaling Technology, 9509S), Caspase-3 (Cell Signaling Technology, 9662S), Cleaved-Caspase-3 (Cell Signaling Technology, 9661S), GAPDH (GNI, GNI4110-GH-M). Secondary antibodies: Sheep anti-rabbit Cy3 or FITC (Jackson ImmunoResearch), Sheep anti-mouse Cy3 or FITC (Jackson ImmunoResearch). Fluorescent probes and dyes: F-actin (Cytoskeleton, CY-SC001), DCFH-DA (Solarbio, D6470), MitoSOX (ThermoFisher, M36008), JC-1 (Cayman, 10009172-100T), DAPI (Sigma), Hoechst 33,342 (Beyotime, C1028). Chemicals: Berberine (Chengdu Mansite Biotechnology Co., LTD, A0151), Mitochondrial ROS scavenger MitoTEMPO (Sigma, SML0737).

## 3 Methods

### 3.1 Cell culture and treatment

Primary VSMCs were isolated from mouse aortic tissue and cultured on six-well plates coated with 1% gelatin, with a silicone elastomer bottom. Cells were maintained in DMEM containing 10% fetal calf serum, 100 μM streptomycin, and 100 U/mL penicillin in a humidified incubator at 37 °C with 5% CO_2_. When cells reached approximately 80% confluence, they were serum-starved for 24 h to induce quiescence and then exposed to cyclic SS using a Flexcell FX-3000 Tension System (Flexcell International Corp., Burlington, NC, USA). This system includes a controlled vacuum unit and a baseplate designed to hold the culture plates (FX3000 AFC-CTL). Cyclic vacuum (15–20 kPa) was applied to the elastomer-bottomed plates at 60 cycles/min with 10% elongation, generating a uniform stretch across the cell monolayer. To examine the effects of BBR and mitochondrial ROS inhibition, cells were pretreated with BBR or the mitochondrial ROS scavenger MitoTEMPO before SS exposure. VSMCs were treated with berberine at 100 μM for 24 h, with an equivalent concentration of DMSO used as the vehicle control. The selected concentration was determined based on previous studies demonstrating the protective effects of berberine in vascular cells within a biologically relevant range while avoiding significant cytotoxicity. Cell viability was further evaluated using [CCK-8 assay], confirming that berberine treatment did not cause apparent cytotoxic effects under the experimental conditions. All experiments were performed using multiple biological replicates from independent VSMC isolations.

### 3.2 Immunofluorescence staining

VSMCs at 50%–60% confluence were serum-starved for 24 h. Cells were washed twice with PBS and fixed with 4% paraformaldehyde (PFA) at room temperature for 20 min. Membranes were permeabilized with 0.3% Triton X-100 in PBS for 10 min and blocked with 5% (w/v) BSA. Cells were incubated with the appropriate primary antibodies at 4 °C overnight, followed by incubation with Cy3-or FITC-conjugated secondary antibodies and an F-actin probe at 37 °C for 2 h in the dark. Nuclei were counterstained with DAPI. Images were captured using a Leica DMI8 fluorescence microscope.

### 3.3 Live-cell fluorescence co-staining

For mitochondrial membrane potential detection, cells were incubated in serum-free medium containing F-actin probe (1 µM), JC-1 (1:100), and Hoechst 33,342 (1X) at 37 °C for 30 min in the dark. For ROS detection, cells were incubated in serum-free medium containing F-actin probe (1 µM), F-actin rearrangement represents cytoskeletal remodeling associated with VSMC phenotypic modulation. DCFH-DA (10 µM), MitoSOX (2 µM), and Hoechst 33,342 (1X) under the same conditions. After incubation, cells were washed once with serum-free medium, and fluorescence images were acquired using a fluorescence microscope.

### 3.4 Western blot analysis

VSMCs at 80% confluence were serum-starved for 24 h, collected on ice with PBS using a cell scraper, and lysed in Buffer A (1 mM Na_3_VO_4_, 2 mM EDTA, 1 mM DTT, 20 mM HEPES, 50 mM β-glycerol phosphate, 1% Triton X-100, 10% glycerol, pH 7.4) supplemented with protease inhibitors (25: 1 ratio) on ice for 2 h. Protein concentrations were quantified using a BCA assay (Shanghai Yamei Biomedical Technology Co., LTD, ZJ101). Proteins were heat-denatured, separated by SDS-PAGE, and transferred to nitrocellulose membranes. Membranes were incubated with specific primary and secondary antibodies, and bands were visualized using an enhanced chemiluminescence (ECL) detection system. Band intensities were quantified with ImageJ software (NIH, Bethesda, MD, USA).

### 3.5 Statistical analysis

Data are presented as mean ± standard deviation (SD). Statistical analyses and graphical representations were performed using ImageJ, GraphPad Prism 7.0, and SPSS 25.0. Groups were compared using one-way analysis of variance (ANOVA). A *p*-value <0.05 was considered statistically significant. Prior to performing one-way ANOVA, data distribution was evaluated using the Shapiro–Wilk normality test, and homogeneity of variance was assessed using Levene’s test. When ANOVA indicated significant differences among groups, appropriate *post hoc* comparisons were performed using Tukey’s multiple comparison test. All quantitative data are presented as mean ± SD (or mean ± SEM, as appropriate), and statistical significance was defined as p < 0.05.

## 4 Results

### 4.1 BBR inhibited SS-induced proliferation and apoptosis of VSMCs

To evaluate the effects of BBR on SS-induced proliferation and apoptosis in VSMCs, immunofluorescence co-staining for F-actin, Ki67, and cleaved caspase-3 was performed. Cleaved caspase-3, a key mediator of apoptotic signaling (Zhang et al., 2019), served as an apoptosis marker. As shown in Figure 1A, BBR significantly inhibited both SS-induced proliferation and apoptosis. Cleaved caspase-3 expression was positively associated with increased F-actin levels under SS conditions, indicating a tendency toward apoptosis. In comparison, VSMCs with lower F-actin expression showed higher Ki67 positivity, highlighting enhanced proliferation (Figures 1A–C). These results suggest that intrinsic cellular heterogeneity among VSMCs determines their differential fate toward survival or apoptosis under SS.

**FIGURE 1:**
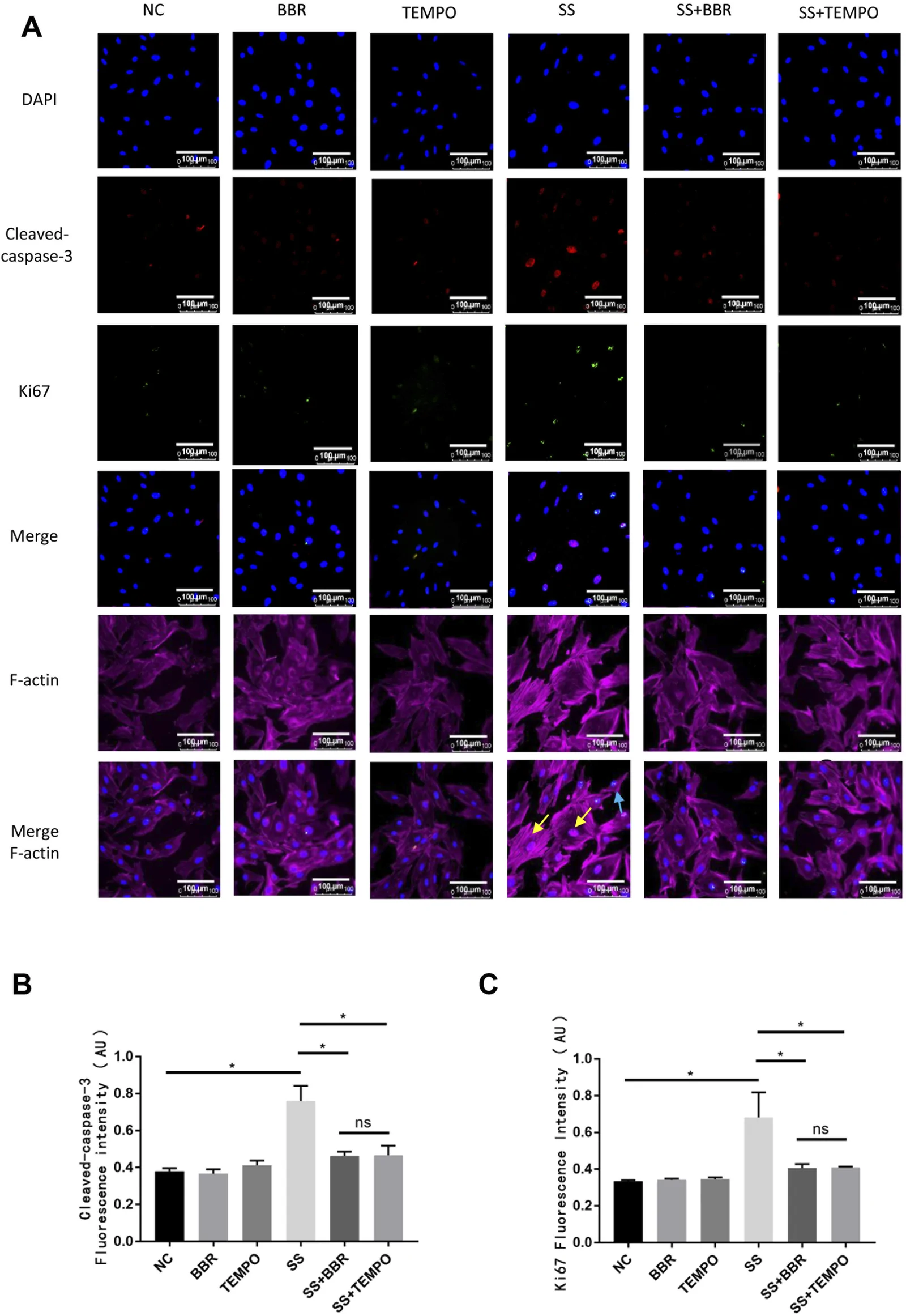
BBR inhibited SS-induced proliferation and apoptosis in VSMCs. **(A–C)** Immunofluorescence co-staining shows that BBR attenuates VSMCs proliferation and apoptosis induced by SS. Yellow arrows indicate apoptotic signaling in VSMCs with high F-actin expression, whereas blue arrows indicate proliferative signaling in VSMCs with low F-actin expression. BBR, berberine; TEMPO, MitoTEMPO (mitochondrial ROS scavenger). *p < 0.05; ns, not significant. Scale bar = 100 μm.

### 4.2 VSMCs with differential F-actin expression respond differently to SS stimulation

As shown in Figure 2, SS significantly increased both total and mitochondrial ROS levels in VSMCs, while BBR treatment decreased ROS production, similar to the effect of a mitochondrial ROS scavenger. VSMCs with high F-actin expression showed well-organized myofilament structures and strong fluorescence intensity after SS stimulation. In comparison, VSMCs with low F-actin expression mainly showed actin monomer distribution, along with lower ROS levels (Figures 2A–C). Furthermore, mitochondrial membrane potential was measured using the JC-1 probe. The results showed that mitochondrial depolarization patterns aligned with ROS production levels (Figures 2D,E). These results suggest that VSMCs with heterogeneous F-actin expression respond differently to SS and that SS causes mitochondrial dysfunction.

**FIGURE 2:**
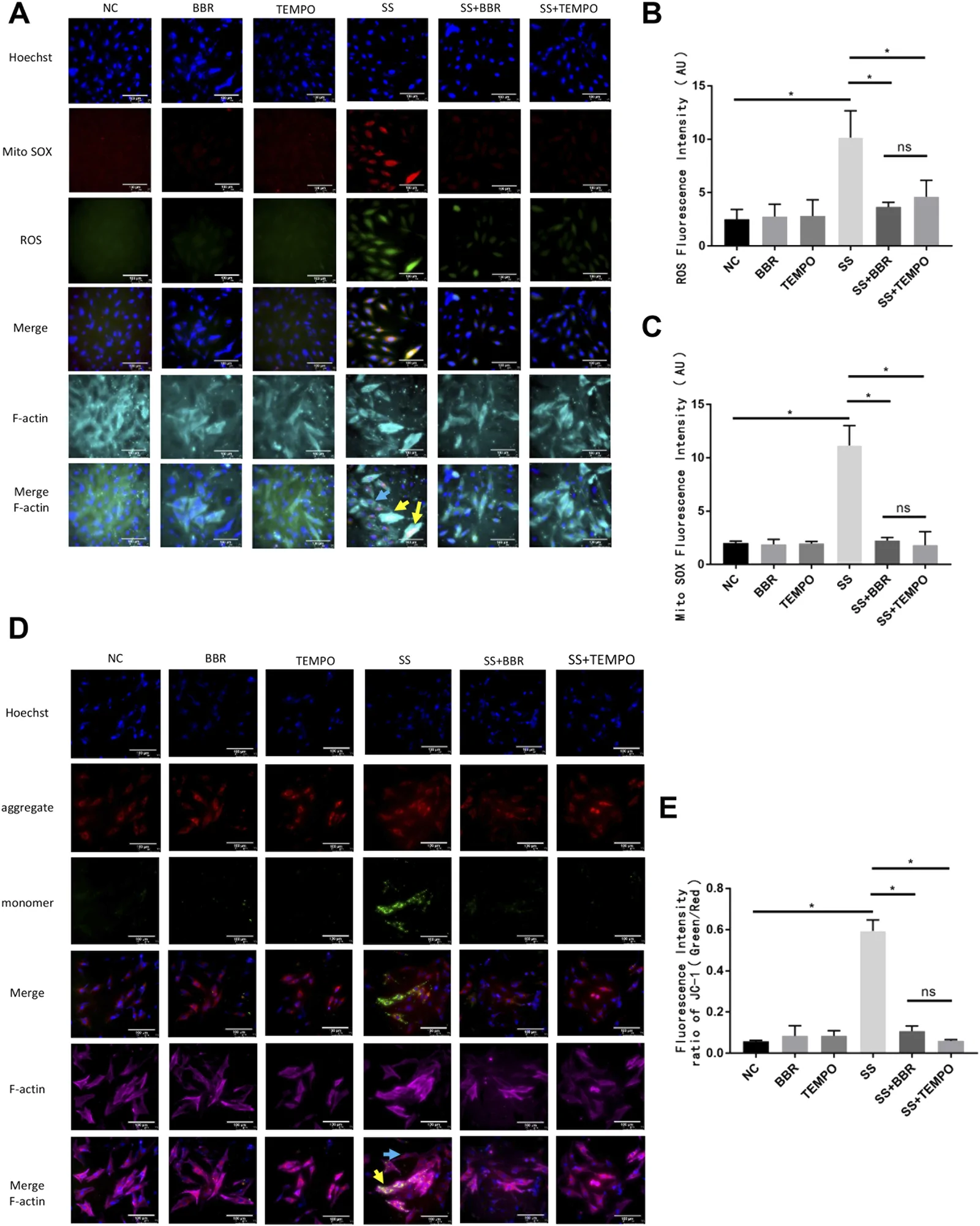
BBR inhibited SS-induced mitochondrial dysfunction in VSMCs. **(A–C)** Stretch stress (SS) increases total and mitochondrial ROS levels in VSMCs. Cells with high F-actin expression show elevated ROS production, whereas cells with low F-actin expression show comparatively lower ROS levels. BBR reduces SS-induced ROS generation. Yellow arrows indicate high ROS levels in VSMCs with overexpression of F-actin, while blue arrows indicate lower ROS levels in VSMCs with reduced F-actin expression. **(D,E)** SS induces differential depolarization of the mitochondrial membrane potential in VSMCs depending on F-actin expression levels. VSMCs with high F-actin expression show more mitochondrial depolarization, whereas those with low F-actin expression show reduced depolarization. Yellow arrows indicate high levels of mitochondrial membrane depolarization, while blue arrows indicate lower levels. BBR, berberine; TEMPO, MitoTEMPO (mitochondrial ROS scavenger). *p < 0.05; ns, not significant. Scale bar = 100 μm.

### 4.3 BBR inhibited SS-induced co-activation of PDI and Drp1 in VSMCs

PDI regulates mitochondrial fission by interacting with Drp1, thus promoting mitochondrial ROS production. PKC-α is also crucial in maintaining the homeostasis between cellular proliferation and apoptosis by modulating oxidative stress pathways (Xiaodong et al., 2023). To explore the association between PKC-α and the PDI/Drp1 signaling pathway, VSMCs were pretreated with the PKC-α inhibitor Go6976 before SS exposure. As shown in Figures 3A–C, both BBR and Go6976 significantly suppressed the oxidation of PDI and the phosphorylation of Drp1, showing the antioxidative effects of BBR. Furthermore, BBR substantially attenuated SS-induced activation of PDI and Drp1 by decreasing mitochondrial ROS production (Figures 3D–F). Immunofluorescence analysis was performed to observe the subcellular localization of PDI and Drp1 (Figures 3G,H). Both PDI and Drp1 were co-localized within the cytoplasm of VSMCs. Moreover, F-actin expression patterns aligned with the distribution of PDI and Drp1, further supporting functional heterogeneity among VSMCs.

**FIGURE 3:**
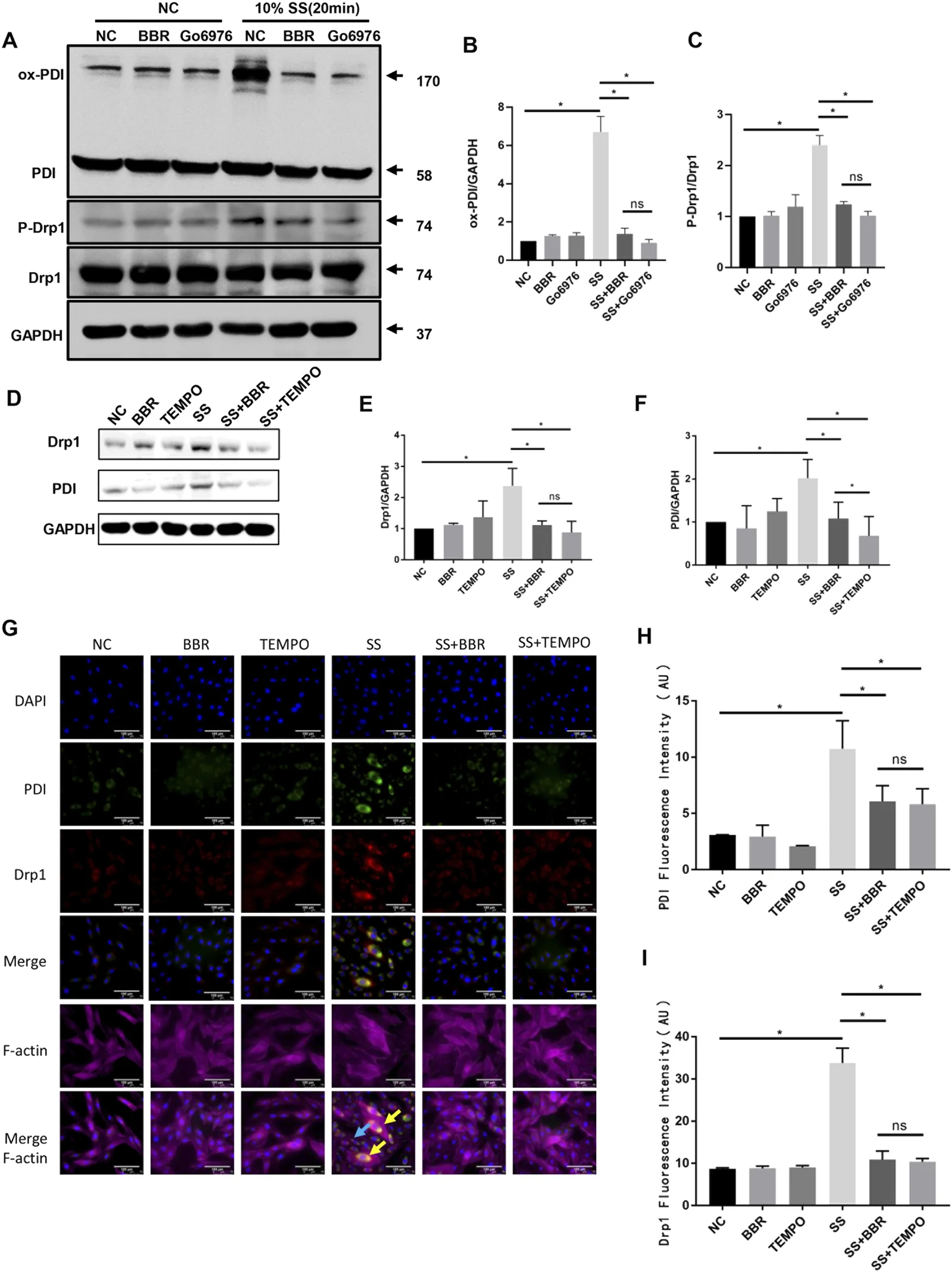
BBR inhibited SS-induced co-expression of PDI and Drp1 in VSMCs. **(A–C)** BBR inhibits the activation of ox-PDI and phosphorylation of Drp1; **(D–F)** BBR reduces SS-induced expression of PDI and Drp1; **(G–H)** Immunofluorescence shows co-localization of PDI and Drp1 with F-actin. Yellow arrows indicate high PDI/Drp1 expression in VSMCs with high F-actin, while blue arrows indicate low expression in VSMCs with low F-actin. BBR, Berberine; TEMPO, Mito TEMPO, mitochondrial ROS scavenger; Go6976, PKCα inhibitor; *, *p* < 0.05, ns, not significant, *p* > 0.05. Scale bar = 100 µm.

### 4.4 BBR inhibited the activation of PKCα/mTOR/p70 and caspase-9/caspase-3 signaling pathway induced by SS

Downstream signaling pathways were analyzed to explore the connection between mitochondrial dysfunction and VSMC proliferation and apoptosis. SS stimulation significantly activated the proliferative mTOR/p70 pathway (Zhang et al., 2020) and the apoptotic caspase-9/caspase-3 pathway (Zeng et al., 2018) in a time-dependent manner, with peak activation at 30 min and 16 h, respectively. BBR effectively reduced the activation of both pathways (Figures 4A–L). These findings show that SS promotes both proliferation and apoptosis through mitochondrial dysfunction-mediated signaling, while BBR broadly inhibits these pathways. SS significantly increased PKC-α activation (Figures 4M–P). Together with the results in Figure 3, these results suggest that SS co-activates PDI and Drp1 *via* PKC-α signaling.

**FIGURE 4:**
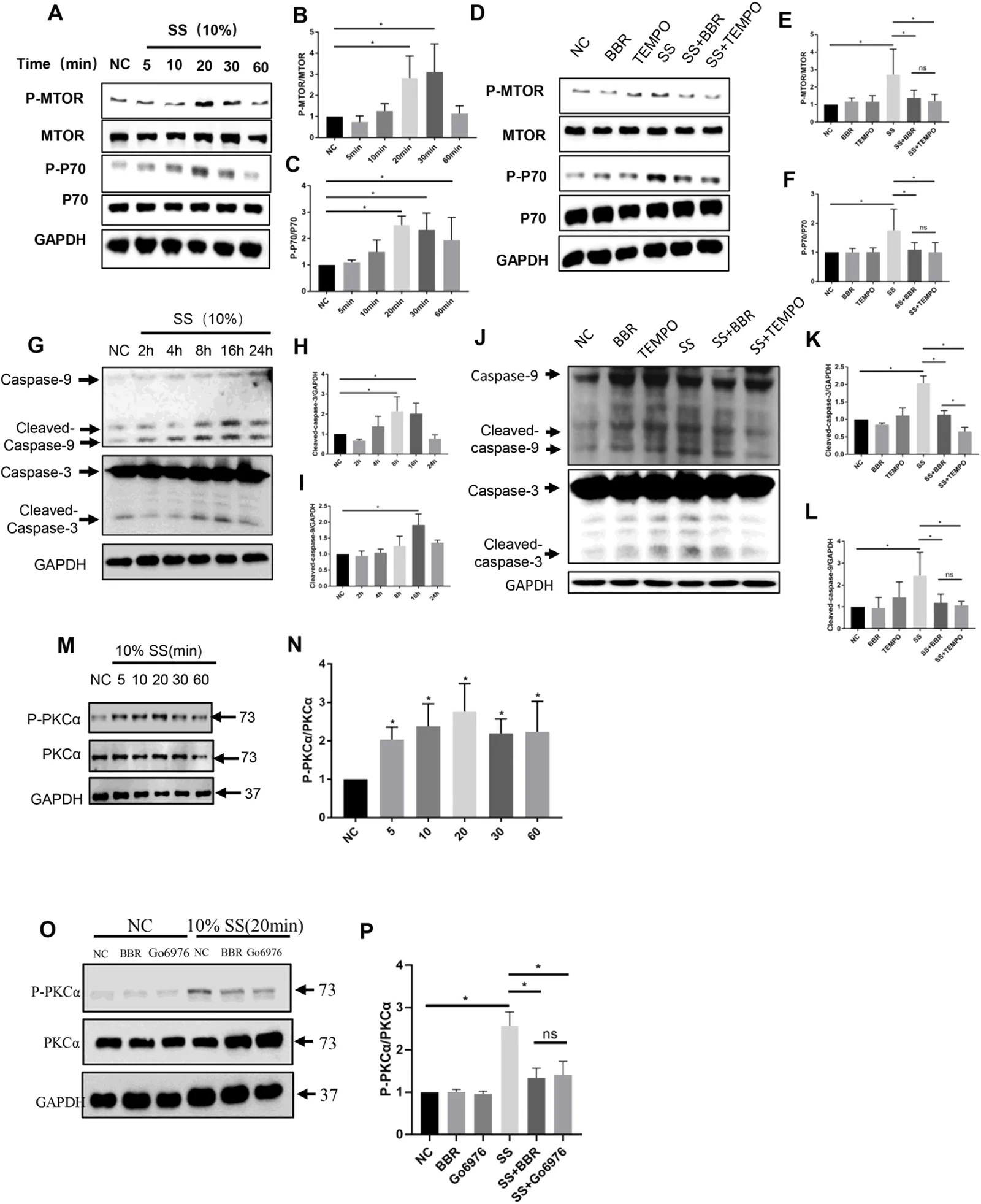
BBR inhibits SS-induced activation of PKCα/mTOR/p70 and Caspase-9/Caspase-3 signaling pathways in VSMCs. **(A–C)** Time-course analysis of SS stimulation shows that mTOR/p70 signaling peaks at 20–30 min **(D–F)** BBR suppresses SS-induced activation of the mTOR/p70 pathway. **(G–I)** Time-course analysis indicates that Caspase-9/Caspase-3 signaling reaches maximal activation at 8–16 h after SS stimulation. **(J–L)** BBR inhibits SS-induced activation of the Caspase-9/Caspase-3 pathway. **(M,N)** SS-induced PKCα phosphorylation peaks at 10–20 min *p < 0.05 vs. NC group. **(O,P)** BBR attenuates SS-induced PKCα phosphorylation. BBR, berberine; TEMPO, MitoTEMPO (mitochondrial ROS scavenger); Go6976, PKCα inhibitor. *p < 0.05; ns, not significant. Scale bar = 100 μm.

### 4.5 PKCα mediated the effects of BBR on the activation of the PDI/Drp1 signaling pathway induced by SS

To further elucidate the regulatory relationship between PKC-α and PDI/Drp1 signaling, VSMCs were treated with the PDI inhibitor bacitracin before SS stimulation. As shown in Figures 5A–F, both BBR and bacitracin significantly reduced SS-induced phosphorylation of Drp1, mTOR, and p70; however, neither treatment affected PKC-α phosphorylation. These results suggest that PKC-α acts upstream of PDI and regulates the downstream Drp1/mTOR/p70 signaling pathway. To validate this pathway, Drp1 expression was silenced with siRNA before SS exposure. *Drp1* knockdown significantly reduced mTOR and p70 phosphorylation, without affecting PKC-α or PDI activation (Figures 5I–O), indicating that Drp1 functions downstream of PKC-α/PDI and regulates mTOR/p70 signaling. These results show that BBR inhibits SS-induced activation of the PKC-α/PDI/Drp1/mTOR/p70 signaling pathway through multi-target regulation, thus suppressing VSMC proliferation. Moreover, PKC-α was found to mediate activation of the apoptotic caspase-9/caspase-3 pathway under SS conditions (Figures 5P–R).

**FIGURE 5:**
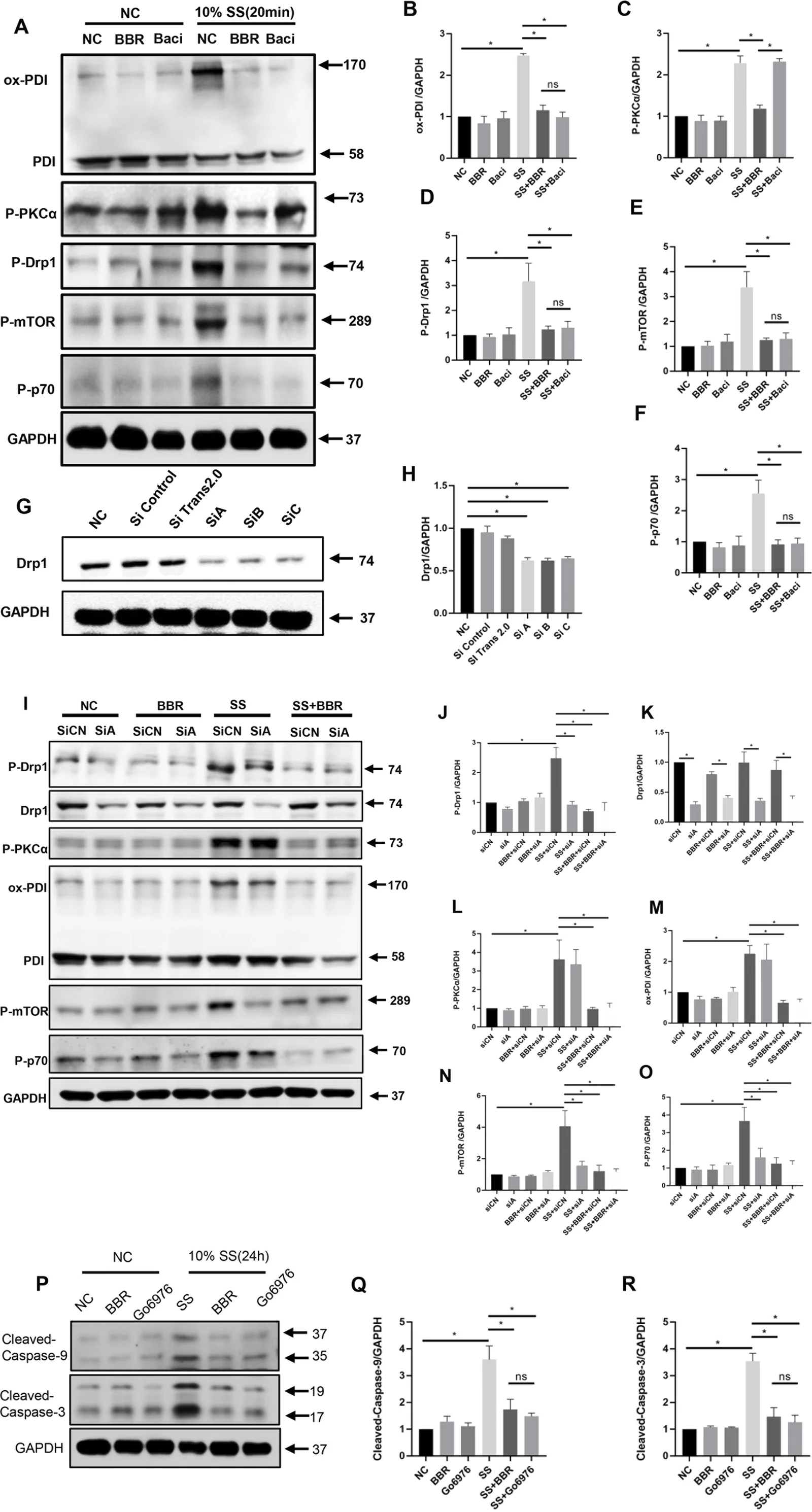
PKCα mediates the effects of BBR on SS-induced activation of the PDI/Drp1 signaling pathway in VSMCs. **(A–F)** BBR inhibits SS-induced activation of the PKCα/PDI/Drp1/mTOR/p70 pathway, whereas the PDI inhibitor bacitracin does not affect PKCα activation. **(G,H)** Efficient knockdown of Drp1 expression by siRNA. **(I–O)** Drp1 silencing does not alter PKCα or PDI activation but suppresses SS-induced mTOR and p70 signaling. **(P–R)** BBR and the PKCα inhibitor Go6976 attenuate SS-induced activation of the Caspase-9/Caspase-3 pathway. BBR, berberine; Baci, bacitracin (PDI inhibitor); Go6976, PKCα inhibitor. *p < 0.05; ns, not significant. Scale bar = 100 μm.

### 4.6 PKCα regulates SS-induced activation of the PDI/mTOR/Cas-9/Cas-3 signaling pathway

To further investigate the relationship between PKC-α and the apoptotic signaling cascade, VSMCs were pretreated with the autophagy inhibitor with effects on the PI3K-related pathway (3-MA) before SS stimulation. As shown in Figures 6A–I, 3-MA did not affect SS-induced activation of the PKC-α/PDI/Drp1 pathway, consistent with previous results. Moreover, inhibition of mTOR did not inhibit activation of the caspase-9/caspase-3 pathway, suggesting that this apoptotic pathway is independent of mTOR signaling. In comparison, treatment with the PDI inhibitor bacitracin significantly decreased SS-induced activation of caspase-9 and caspase-3 (Figures 6J–L), indicating that PDI acts upstream of the apoptotic signaling cascade.

**FIGURE 6:**
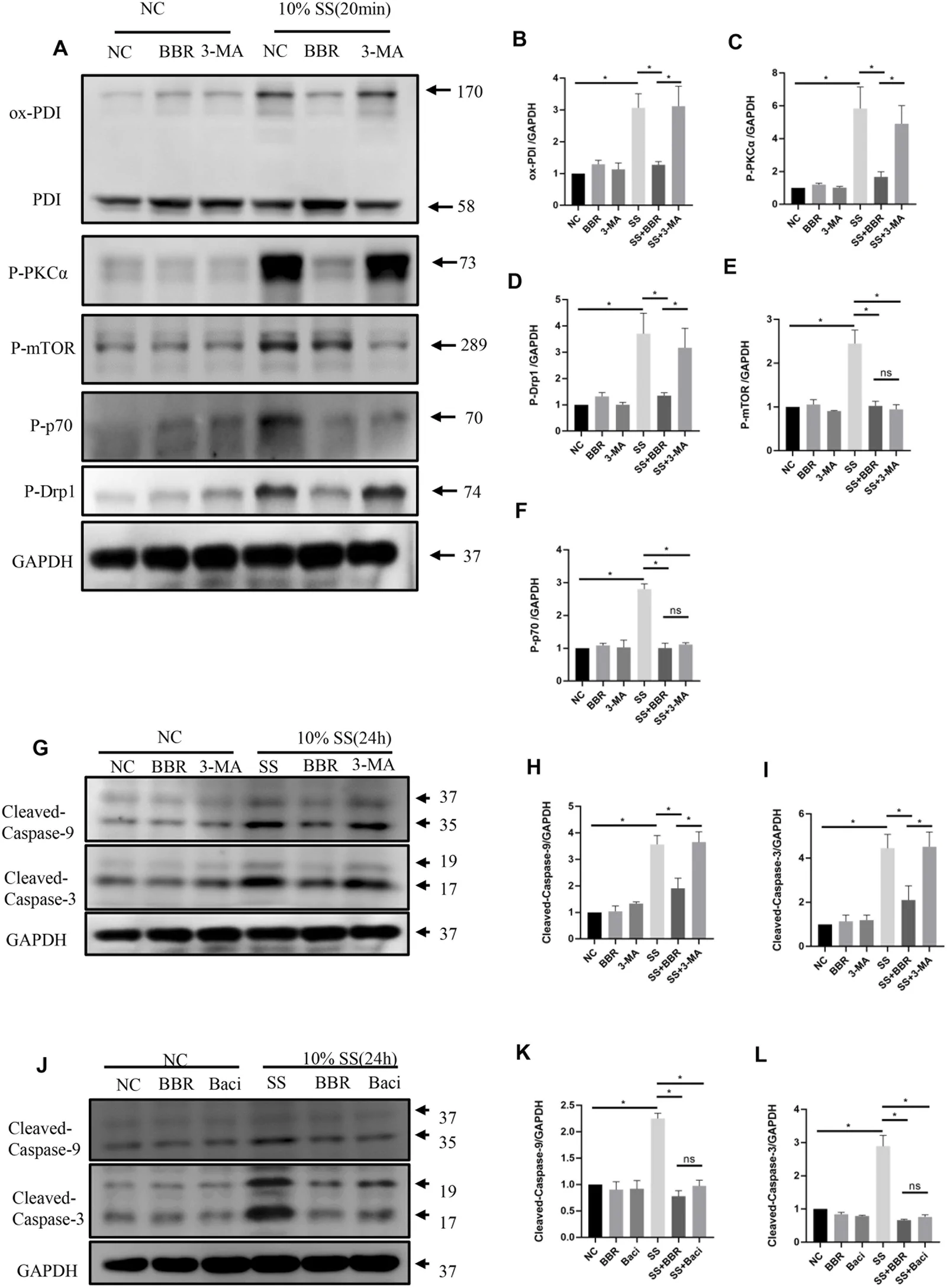
PKCα mediates the effects of BBR on SS-induced activation of PDI/mTOR and Caspase-9/Caspase-3 signaling pathways in VSMCs. **(A–F)** BBR inhibits SS-induced activation of the PKCα/PDI/Drp1/mTOR/p70 pathway, whereas the mTOR inhibitor 3-MA does not affect PKCα/PDI/Drp1 activation. **(G–I)** BBR suppresses SS-induced activation of the Caspase-9/Caspase-3 pathway, while 3-MA shows no inhibitory effect. **(J–L)** BBR and the PDI inhibitor bacitracin attenuate SS-induced activation of the Caspase-9/Caspase-3 pathway. BBR, berberine; 3-MA, mTOR inhibitor; Baci, bacitracin (PDI inhibitor); Go6976. *p < 0.05; ns, not significant. Scale bar = 100 μm.

### 4.7 mTOR-mediated SS-induced proliferation, while Drp1 regulates proliferation and apoptosis

Previous findings have shown that SS induces heterogeneous ROS production in VSMCs (Lee and Kim, 2018); however, its role in regulating proliferation and apoptosis remains to be elucidated. As shown in Figures 7A,B, both the mTOR inhibitor 3-MA and the PDI inhibitor bacitracin significantly suppress SS-induced VSMC proliferation. Moreover, silencing *Drp1* with siRNA significantly reduced SS-induced activation of cleaved caspase-9 and cleaved caspase-3 (Figures 7C–F). Co-staining of Ki67 and TUNEL showed that *Drp1* knockdown significantly decreased both proliferative and apoptotic cell populations under SS conditions (Figures 7G–I). These results indicate that mTOR mainly mediates SS-induced proliferation, while Drp1 regulates both proliferation and apoptosis in response to SS. As shown in Figure 8.

**FIGURE 7:**
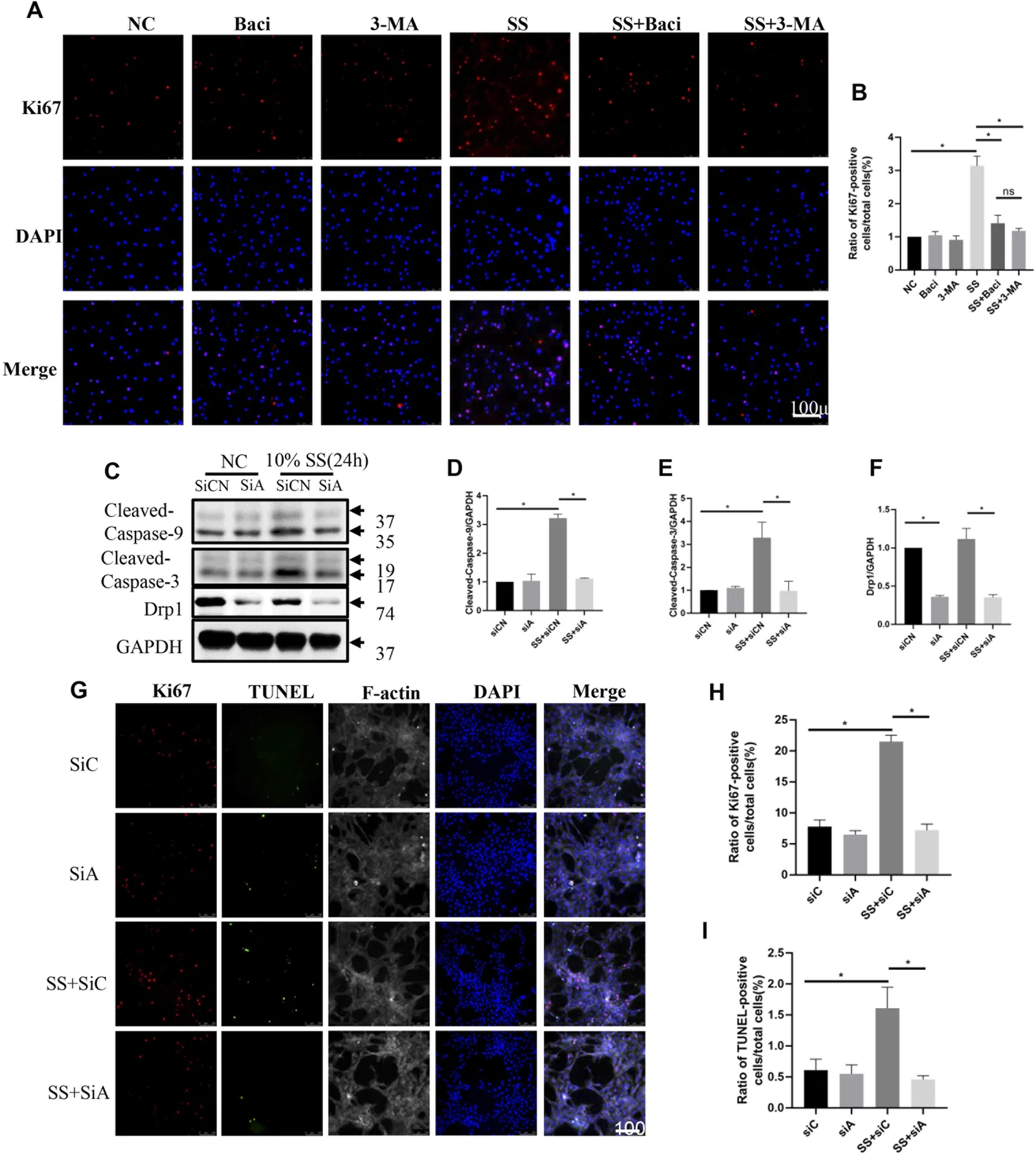
mTOR mediates SS-induced proliferation, while Drp1 regulates both proliferation and apoptosis in VSMCs. **(A,B)** Inhibition of PDI and mTOR reduces SS-induced VSMC proliferation. **(C–F)** *Drp1* knockdown by siRNA suppresses SS-induced activation of Caspase-9/Caspase-3. **(G–I)** *Drp1* silencing attenuates proliferation and apoptosis of VSMCs under SS conditions. BBR, berberine; 3-MA, mTOR inhibitor; Baci, bacitracin (PDI inhibitor). *p < 0.05; ns, not significant. Scale bar = 100 μm.

**FIGURE 8:**
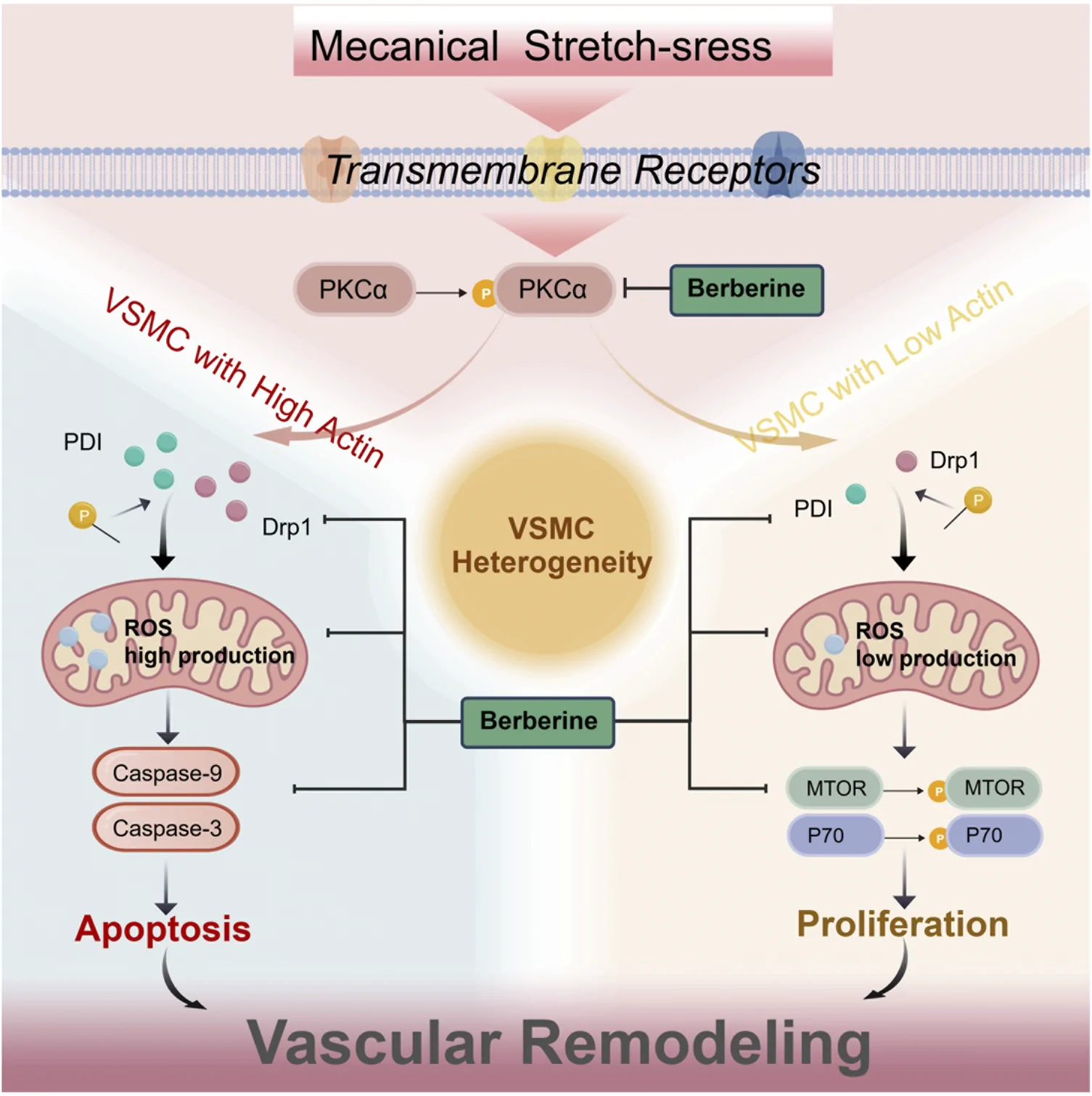
Schematic illustration of SS-induced heterogeneous regulation of proliferation and apoptosis in VSMCs and the inhibitory effects of BBR. Due to the heterogeneity of actin expression in VSMCs, when Stretch stress (SS) activates transmembrane receptors and leads to the phosphorylation of PKCα, in VSMCs with higher actin expression levels, the activation of PKCα will upregulate the expression and phosphorylation of PDI and Drp1, thereby increasing the production of mitochondrial ROS and activating the Caspase-9/Caspase-3 pathway, inducing cell apoptosis. At the same time, in VSMCs with lower actin expression levels, the activation of PKCα will lead to a lower expression and phosphorylation of PDI and Drp1, leading to reduced mitochondrial ROS production and activation of the mTOR/p70 pathway, promoting cell proliferation. The simultaneous increase in proliferation and apoptosis contributes to vascular remodeling. BBR reduces both processes through multi-target inhibition of these signaling pathways.

## 5 Discussion

This study investigated the mechanisms by which SS regulates VSMC proliferation and apoptosis *via* the ROS/PDI/Drp1 signaling pathway. Our findings demonstrate that VSMCs demonstrate intrinsic heterogeneity, characterized by differential expression of F-actin at the single-cell level. Exposure to SS led to variable ROS production among VSMCs. Further examination revealed that (1) SS stimulation induced elevated ROS accumulation in VSMCs with high F-actin expression, whereas cells with low F-actin expression showed comparatively lower ROS levels; this effect was effectively attenuated by BBR; (2) SS promoted mitochondrial dysfunction through upregulation of the PKCα/PDI/Drp1 signaling axis; and (3) SS simultaneously induced VSMC proliferation and apoptosis by activating the downstream mTOR/p70 proliferative pathway and the caspase-9/caspase-3 apoptotic pathway, both of which were significantly inhibited by BBR. These results underscore the crucial role of mechanical stress in vascular remodeling. Furthermore, by incorporating F-actin and ROS production as key factors, this study establishes a mechanistic link between VSMC heterogeneity and SS-induced proliferation and apoptosis *in vitro*. These insights provide a potential basis for developing therapies to address vascular remodeling related to hypertension.

Previous studies have shown that VSMCs show distinct phenotypic and functional responses when exposed to different mechanical stimuli. For example, Liu et al. reported that hypertensive mechanical stress promotes VSMC phenotypic changes through activation of the YAP/TAZ signaling pathway (Wang et al., 2018). In this study, we further link F-actin expression, ROS production, mitochondrial function, and cell fate determination in VSMCs, providing direct evidence of VSMC heterogeneity. These results are in good agreement with the observations of Yang et al. (Yang et al., 2024)., who showed that F-actin polymerization is crucial for mediating VSMC phenotypic transformation. ROS acts as crucial intracellular signaling molecules, with their production closely linked to cytoskeletal dynamics. Previous studies have shown that ROS generation promotes F-actin polymerization, as demonstrated by Ishimoto et al. (Ishimoto and Mori, 2021). Moreover, increased mechanical stress has been reported to enhance F-actin cytoskeletal remodeling and ROS production, contributing to extracellular matrix damage in human chondrocytes (Lee et al., 2021). These results indicate a strong connection between cytoskeletal organization and oxidative stress. Mitochondrial dysfunction and excessive ROS production are key features of cardiovascular diseases. Our results showed that higher ROS levels are related to significant mitochondrial membrane depolarization in VSMCs, indicating impaired mitochondrial function. Previous research has shown that loss of Drp1 function decreases calcium accumulation in both the cytoplasm and mitochondria during cellular injury, resulting in reduced ROS production and disrupted F-actin organization, ultimately compromising tissue repair processes (Zeng et al., 2022). Furthermore, recent evidence suggests that PDI may act as a nitric oxide donor for Drp1, facilitating its S-nitrosylation and increasing phosphorylation at Ser616, thus promoting mitochondrial fission and increasing mitochondrial ROS production (Lee and Kim, 2018). Jin et al. (Jin et al., 2021). further observed that Drp1-dependent mitochondrial fission plays a crucial role in vascular remodeling. This study shows that SS-induced mitochondrial ROS bursts are closely associated with Drp1-mediated mitochondrial fission. Moreover, PDI, as an upstream regulator of Drp1, showed expression patterns aligned with F-actin levels, suggesting that cytoskeletal dynamics may affect mitochondrial function by regulating PDI activity.

This study shows that SS promotes VSMC proliferation by activating the PKCα/PDI/Drp1/mTOR/p70 signaling pathway. The mTOR/p70 pathway, a key downstream component of mitochondrial oxidative stress, is involved in regulating cell proliferation (Kurgan et al., 2017). Previous research indicates that platelet-derived growth factor-BB (PDGF-BB) causes VSMC dedifferentiation, proliferation, and migration by increasing ROS production and activating the ROS/NF-κB/mTOR/p70S6K signaling pathway (Lu et al., 2018), which aligns with the current findings. Moreover, the role of mTOR signaling in cell proliferation has been widely confirmed across various cell types. For example, Zhao et al. reported that TGF-β promotes pericyte proliferation and phenotypic transition in subretinal fibrosis through activation of the Akt/mTOR pathway (Zhao et al., 2022). Our results further support these observations by confirming the involvement of mTOR signaling in SS-induced VSMC proliferation (Zhao et al., 2022). Mitochondrial-mediated apoptosis plays a key role in the development of AS (Cheng et al., 2023). Activation of the caspase-9/caspase-3 cascade is a crucial step in mitochondrial-dependent apoptosis. It is well known that oxidative stress can induce caspase-9 activation *via* mitochondrial membrane depolarization, leading to apoptosis in cardiomyocytes and neuronal cells (Krajewski et al., 1999). Our results show that SS induces apoptosis by increasing mtROS production and activating the caspase-9/caspase-3 pathway, especially in VSMCs with higher F-actin expression.

PDI has been implicated in cardiovascular pathology by modulating oxidative stress and mitochondrial dynamics, although its direct role in cardiovascular tissues remains an emerging research area (Jin et al., 2021). Under pathological stimuli, PDI-mediated redox modification may promote Drp1 activation, while PKCα serves as an upstream kinase that enhances Drp1 Ser616 phosphorylation and mitochondrial translocation, leading to excessive mitochondrial fission. Activation of the PKCα/Drp1 axis induces mitochondrial fragmentation, mitochondrial ROS accumulation, cardiomyocyte injury, cardiac fibroblast activation, and vascular remodeling, thereby contributing to myocardial ischemia/reperfusion injury, cardiac fibrosis, and cardiovascular disease progression. Targeting the PDI/PKCα/Drp1 pathway may represent a potential therapeutic strategy by restoring mitochondrial homeostasis and reducing oxidative damage in cardiovascular disorders (Tseng et al., 2019; Kaur et al., 2024).Our previous researches demonstrated that proliferation and apoptosis exist at the same field of the VSMCs under SS (Ping et al., 2017; Wang L. et al., 2020; Ping et al., 2015), the mechanism is different signalings that control the proliferation and apoptosis, which is proved in this study. Previous reports seldom show the contradictory results such as proliferation and apoptosis occur in the same field under the same stimuli, but we tried to uncover the deep mechanism, which VSMCs proliferation is induced by mTOR/p70, and VSMCs apoptosis is induced by caspase-9/caspase-3 pathway.

BBR, a pleiotropic natural compound, has gained significant attention for its potential therapeutic role in cardiovascular diseases (Feng et al., 2019). Besides its well-known lipid-lowering and anti-inflammatory effects, emerging evidence highlights its antioxidant and mitochondrial-protective functions. For example, Shen et al. showed that BBR improves septic cardiomyopathy by maintaining mitochondrial function and activating Notch1 signaling in cardiomyocytes (Shen et al., 2024). Similarly, Wang et al. showed that BBR inhibits ferroptosis and stabilizes atherosclerotic plaques by modulating the NRF2/SLC7A11/GPX4 pathway (Wang et al., 2024). In our previous published paper, BBR was used to explore its effect on the target, but only proved the overall effects, the corresponding signalings are not clear, so in the present study BBR was also applied to verify its effects on the PKCα/PDI/Drp1 signaling pathway and mtROS production, to provide more evidence on BBR’s effects on vascular diseases and to explore more similar drugs.

This study shows that BBR effectively inhibits SS-induced activation of the PKCα/PDI/Drp1 signaling pathway and reduces mtROS production, thus exerting dual suppression of proliferative and apoptotic mechanisms. These results further highlight the vital role of BBR in modulating mechanical stress-induced vascular remodeling and reveal its potential as a therapeutic option for managing AS. Although our findings suggest that berberine may exert vascular protective effects through regulation of the ROS/PDI/Drp1-mediated mitochondrial pathway, these conclusions should be interpreted cautiously. The present study provides mechanistic evidence from *in vitro* models, and further validation using *in vivo* disease models, pharmacokinetic evaluation, dose optimization, and clinically relevant endpoints is required before considering berberine as a potential therapeutic strategy for vascular disorders. We acknowledge that several important aspects, including *in vivo* validation using appropriate animal models, pharmacokinetic/pharmacodynamic (PK/PD) characterization, dose-response relationships, bioavailability, and clinically relevant vascular outcomes, require further investigation before the therapeutic potential of berberine can be fully established. As a naturally occurring compound, berberine exhibits complex pharmacological properties, including relatively low oral bioavailability and extensive metabolism, which are important considerations for its clinical translation.

## 6 Limitations

Despite these promising findings, this study also has some limitations. First, the therapeutic efficacy of BBR requires further *in vivo* validation, particularly regarding optimal dosing and administration methods. Secondly, the specific molecular mechanisms underlying PDI function in VSMCs require further study, ideally using conditional gene-knockout models. Thirdly, there is no animal experiments and clinical speciments. Future research will focus on addressing these issues. Fourthly, reactivation or overexpression of Drp1, constitutively active PKCα, or genetic modulation of PDI would help determine whether this pathway is required and sufficient for the observed effects.It should be noted that berberine is a pleiotropic bioactive compound that regulates multiple signaling pathways, including AMPK, SIRT1, NRF2, NF-κB, and mitochondrial homeostasis. Therefore, although our findings highlight the involvement of the ROS/PDI/Drp1 axis in mediating the protective effects of berberine, other signaling mechanisms may also contribute to its biological effects. Another one is that F-actin remodeling reflects cytoskeletal organization rather than serving as a definitive marker of VSMC phenotypic identity future studies incorporating established contractile and synthetic phenotype markers, such as α-SMA, SM22α, calponin, and osteopontin, together with additional functional and molecular characterization, would provide a more comprehensive definition of VSMC phenotypic heterogeneity.

## 7 Conclusion

In conclusion, this study shows that SS induces different cellular responses in VSMCs depending on F-actin levels. In VSMCs with high F-actin expression, SS promotes mitochondrial fission through the PDI/Drp1 pathway, resulting in excessive ROS production, mitochondrial membrane depolarization, and activation of the PKCα/PDI/Drp1/caspase-9/caspase-3 apoptotic cascade. In comparison, VSMCs with low F-actin levels exhibit lower ROS levels and tend to activate the PKCα/PDI/Drp1/mTOR/p70 pathway, supporting cell growth. BBR effectively inhibits both apoptotic and proliferative signaling pathways across heterogeneous VSMC populations. These results provide novel insights into the mechanisms behind mechanical stress–induced vascular remodeling in hypertension and identify possible therapeutic targets for AS and related vascular diseases. Advanced live-cell super-resolution microscopy and genetically encoded ROS biosensors should be applied to capture real-time, single-cell spatiotemporal dynamics of F-actin remodeling, Drp1-mediated fission, and mtROS bursts, thereby establishing a causal link between cytoskeletal heterogeneity and fate determination.

## Data Availability

The data that support the findings of this study are available from the corresponding author upon reasonable request.
